# The Use of Electronic Games in Therapy: a Review with Clinical Implications

**DOI:** 10.1007/s11920-014-0520-6

**Published:** 2014-10-14

**Authors:** H. Lynn Horne-Moyer, Brian H. Moyer, Drew C. Messer, Elizabeth S. Messer

**Affiliations:** 1 Medaille College, 18 Agassiz Circle, Buffalo, NY 14214 USA; 2 Electronic Gaming Therapy, Inc., 8616 Main Street, Suite 4, Williamsville, NY 14221 USA

**Keywords:** Psychotherapy, Electronic games, Serious games, Cognitive behavior therapy, Group therapy

## Abstract

Therapists and patients enjoy and benefit from interventions that use electronic games (EG) in health care and mental health settings, with a variety of diagnoses and therapeutic goals. We reviewed the use of electronic games designed specifically for a therapeutic purpose, electronic games for psychotherapy (EGP), also called serious games, and commercially produced games used as an adjunct to psychotherapy, electronic games for entertainment (EGE). Recent research on the benefits of EG in rehabilitation settings, EGP, and EGE indicates that electronic methods are often equivalent to more traditional treatments and may be more enjoyable or acceptable, at least to some consumers. Methodological concerns include the lack of randomized controlled trials (RCT) for many applications. Suggestions are offered for using EG in therapeutic practice.

## Introduction

The first commercially successful electronic games (EG) were developed in the 1970s for entertainment and played in arcades, but they soon found their way into homes [1]. Almost simultaneously, health and mental health care providers started using computer and video games as part of therapy [2] and others began developing EGs for psychotherapy (EGP) [3]. Recent estimates of the prevalence of computer and video game play in America range from 59 % [4] to 63 % [5]. Current technology provides therapists with many new opportunities of intervening with our clients. With smart devices that are carried with us, using the Internet, e-mail, and social media, we are able to gather information and disseminate information in ways that are less time-consuming, more interesting, and more readily available. Examples include online forms, multimedia-presented decision trees, and long distance and/or virtual modes of communication. With advanced graphic and audio-visual performance, we are able to create virtual multisensory environments and audio-visual representation of our imaginary worlds.

We review the use of EG in therapy, both electronic games for psychotherapy (EGP), also called serious games, developed primarily for therapeutic purposes, and electronic games for entertainment (EGE), also called off-the-shelf games, developed primarily for leisure purposes but used as a therapeutic tool. Preliminary evidence supports the use of these methods, although more empirical work is needed. Different approaches to game use and development can be undertaken to devise interventions, but EGP and EGE have potential to be applied to a wide range of health and mental health issues and settings.

### Electronic Games for Health-Related Behaviors

EGP and EGE have been used in health promotion and to improve physical and psychosocial functioning of patients. Both types of games may be used to increase motivation, attention/engagement, knowledge, or physical efficacy; to allow for physical activity, practice, or immediate feedback; and to provide therapeutic imagery and emotional expression. Comprehensive reviews indicate that EGP have been used successfully to improve diet and physical activity in children, and EGE for chemotherapy-related nausea, preoperative anxiety, fitness, physical therapy, and cognitive rehabilitation [6, 7••].

EGP allow for tailoring game design and interfaces to specific needs of patients [8]. For example, Sajjad et al. [9••] describe the use of imagery in a first-person shooter game to allow patients with brain tumors to experience an imagined immune response to the tumors. In a randomized clinical trial (RCT), significant reductions were noted by the authors in depression, anxiety, anger, and disruptive behavior and increases in self-concept, with significant advantages of the intervention over the control in all areas.

EG which have gross motor interfaces, such as the Dance Dance Revolution Dance Mat, WiiMote, and Xbox Kinect, provide physical challenge and a greater variety of physical operations when compared to games with conventional controllers. Use of EG with gross motor interfaces has become one of the standard treatments in rehabilitation therapies to increase fitness, and sedentary games increase health-related knowledge and encourage pro-health behaviors [10•]. Physical risks of all EG include seizure and muscle strain [11]. They have been used with demonstrated efficacy with children [12] and adults, including elderly adults, in community [13], rehabilitation, and long-term care settings [14] to increase overall physical fitness [15•] and physical and functional capabilities, reduce pain [14], and improve aspects of cognitive function, with diagnoses including traumatic brain injury [16•], stroke, Huntington’s disease [17•], and multiple sclerosis [18].

Although a number of investigations consist of pilot and feasibility studies [8, 10•, 12, 15•, 16•], EG interventions have also been found to improve psychosocial functioning with medical populations, including reducing disfigurement-related distress [19], stress management [20], self-confidence [21•], socialization [22••, 21•], and quality of life [21•]. One pilot study [23•] failed to find these effects, however, and in one study [15•], benefits of walking outside on well-being were superior to those associated with Wii Fit activity, although both groups improved. When using computerized cognitive behavior therapy (CCBT) with medical patients, some subjects found the intervention too impersonal [24]. GAMELAB [22••], a protocol for group therapy tailoring different aspects of game play to individual patient goals, is an example of using EG in a group format with children as treatment for acquired brain injury.

Although outcomes are encouraging using EG for cognitive stimulation and retraining, Bisoglio et al. [25••] urge caution in concluding that cognition is meaningfully enhanced by various types of video games, because insufficient rigor has been applied to the investigations. In particular, Bisoglio et al. assert that magnitudes of effect, expectancy effects, statistical interactions, and causal models should be examined. These concerns should be noted overall when evaluating the potential for EG in mental health and health care settings.

### Electronic Games Developed Primarily for Psychotherapy

Most approaches to incorporating technology into the therapist’s office use games or programs specifically designed to serve one or more therapeutic needs. Computer-assisted cognitive behavior therapy (cCBT) includes computer-delivered information and interventions designed to implement established treatment models. In some cases, a specific game is developed or the interventions exhibit a game-like quality, but some elements may be less interactive, such as video demonstrations or relaxation inductions. For example, online self-help for anxiety disorders [26] and handheld devices with diaries and prompting for individuals with obsessive compulsive disorder have been shown to facilitate CBT [27].

In a systematic review of the use of technology in treating various anxiety disorders and depression, Newman et al. [28] concluded that computer-assisted treatment is effective overall and suggested that compliance might be maximized through more therapist exposure. Mohr et al. [29] remarked that the “serious game movement” is finding “entertaining games” being designed for therapeutic purposes, especially in the field of mental health (EGP). In a meta-review of the use of EGP in treating depression [30], support was found for the use of EGP, specifically MoodGYM, Beating the Blues, and Colour Your Life, in enhancing CBT for depression. They also suggested EGP could be cost-effective for therapists.

In a similar review [31], MoodGYM was compared with two other games, Beating the Blues and Good Days Ahead. Good Days Ahead incorporates moderate therapist involvement, whereas the other two games had little to none, and had been compared to an established intervention, representing treatment as usual (TAU). Investigations of the other programs employed a waitlist or other non-equivalent controls. These concerns about methodology are consistent with many in virtual reality (VR) and EGP research, the absence of controlled clinical trials and non-significant findings in comparisons with more traditional techniques. Merry et al. [32] used an RCT approach to look at the effectiveness of SPARX, an EGP aimed at adolescents struggling with depression. SPARX is a self-directed game in which clinicians provide initial contact and a phone follow up after a month, and clients are encouraged to contact the therapist if no improvement is noted. SPARX was found to be effective but not superior to TAU, as demonstrated by a decrease in depressive symptoms by 30 % on a children’s depression rating scale. Although perhaps not a true EGP, a pilot study using a game-like presentation of cCBT for anxiety in children demonstrated changes in anxiety and remission of symptoms [33]. No control group was used.

In addition to anxiety and mood disorders, EGP have been developed to enhance psycho-education, attitude change, relaxation, pain management, social skills, problem-solving skills, emotional modulation, self-control skills, motivation, and therapist-client interaction [34]. Of 11 EGP, five were found to have been systematically assessed for effectiveness: Re-Mission, Personal Investigator, Treasure Hunt, Play Attention, and one unnamed game. The authors concluded that EGP can be effective in increasing compliance, learning, and behavior and affective change [34].

PlayMancer, an EGP designed to help individuals with impulse-related disorders, was found to be well accepted by the users, seemed to be a good format in which to work on attitudes and emotions, and to “make it feasible to apply techniques that tend to be difficult to apply … such as controlled intensive exposure, immediate positive and negative reinforcing, complex biofeedback approach, and real time monitoring of physiological-emotional reactions” [35•, p. 371]. Preliminary results indicated that PlayMancer may facilitate clinical improvement.

Virtual reality exposure therapy (VRET) uses VR to expose clients to sources of stress and/or anxiety in a manner that is realistic enough to be visceral within the relative safety of the therapist’s office. Opris et al. [36] performed a quantitative meta-analysis comparing treatment of anxiety disorders using VRET, traditional CBT, and traditional behavioral therapy (BT). Anxiety disorders examined included fear of flying, panic disorder/agoraphobia, social phobia, arachnophobia, acrophobia, and PTSD. The eight studies of comparing VRET to waitlist control at post treatment demonstrated significant improvement for both CBT and BT, and medium to large effect sizes.

In a systematic review of ten experimental studies using VRET in combination with CBT in the treatment of PTSD [37], VRET was found to be at least as useful as traditional exposure therapy. Methodological concerns of the studies included small sample size and concerns about randomized treatment assignment. The authors suggested that VRET might be of particular value with patients who respond better to the more immersive qualities of VR. In a more recent review of RCTs to explore the effectiveness of VRET in the treatment of PTSD [38•], VRET provided “a robust positive effect” similar to other “exposure-based interventions for PTSD” despite low sample sizes and some challenges in standardization. Although the authors suggest that VRET might provide treatment in a less stigmatizing manner and a more controlled yet immersive delivery than traditional exposure, they suggested that this has not been verified experimentally.

In a line of research exemplifying the diffuse boundary between EGP and EGE, some researchers are using off-the-shelf gaming hardware such as the Xbox Kinect to measure body movement in a virtual reality world, laptop computers with built-in webcams to assess facial movement and to explore expression of emotions within an EGP as treatment for pain-related fear and disability in chronic pain [39•], and facial recognition for children with autism spectrum disorder [40, 41•]. Avatars combined with voice alteration programs are being used to assist an individual suffering from auditory hallucinations by offering a visual and interactive perspective on their hallucinations [42•].

Goh et al. [43] recommended that game designers take into account relevant attributes of the client; gender, age, developmental sophistication, ethnicity, and social economic status affect the individual's response to the EG; diagnoses and treatment objectives guide the style of the EG to be designed. For example, specific treatment objectives such as attention/focus may be aligned with a question-and-answer style game or likewise behavior modification and communication with simulation games and virtual reality [43]. Other examples of treatment objectives were control, reading/learning facts, sensory response, and social skills. These had been aligned to problem-solving, drill and practice, virtual reality, matching games, and virtual reality genres, respectively. Other points of interest included degree of realism/level of immersion, management of goal direction, and motivation/challenge in game play. Although the writers offered research examining these various characteristics of game design, they caution that much of the research has focused on games individually and have not addressed the overall constructs of game design as they apply to all games. The writers also point out that current research seems to focus less on the effectiveness of the games and more on playability and acceptability.

### Psychotherapeutic Use of Electronic Games Developed Primarily for Entertainment

Although games and other electronic interventions may be designed for specific therapeutic purposes, games that are commercially available provide an incredible variety of interfaces and experiences that can be used as an adjunct to therapy. In fact, despite concerns about their use, EGE have been shown to have a number of positive effects. The first study to discover the positive effects of playing EGE included commercially produced games as a control for biofeedback [44]. Playing EGE was as effective as biofeedback for reducing impulsivity, increasing personal influence, and improving one’s self-concept. Recent research and reviews of EGE identified four general areas of beneficial effects: cognitive, motivational, emotional, and social [45••, 25••]. Benefits included increased speed and accuracy of attention and visual special abilities improved learning and memory, executive functions, problem-solving skills and creativity. Motivational benefits included improved diligence and persistence. Emotional benefits of using EGE included improved mood or increases in positive emotion and adaptive regulation strategies for managing negative emotions like anger, anxiety, and sadness. Finally, Granic et al. [45••] noted the social benefits of EGE included increased cooperation, support, and helping behaviors and civic engagement.

EGE has been investigated as an adjunct to individual and group psychotherapy, primarily in behavioral or talk therapy with children or adults with developmental delays. A variety of commercially produced games are appropriate to use, and Granic et al. [45••] provided a helpful framework for describing games along two general dimensions: level of complexity and extent of social interaction. Any game may be classified as more or less social and more or less complex along the two axes, in order to determine how they might facilitate particular therapeutic goals (Table 1).

**Table 1:** Examples of games designated by level of complexity and social involvement

Designation	Game style	Game examples
Complex and social	Multiplayer shooter	Halo, Call of Duty
	Multiplayer action-adventure	Grand Theft Auto, Assassins Creed
	On-line role playing	Runescape, World of Warcraft
	Multiplayer sandbox	Minecraft, Half-Life 2
Complex and nonsocial	Solo role playing	Skyrim, Final Fantasy
	Solo sport	FIFA, NHL14
	Solo fighting	Street Fighter, Mortal Kombat
Simple and social	Racing	Mario Kart, Need for Speed
	Rhythm	Rock Band, Guitar Hero
	Party	Wii Party, Mario Party
Simple and nonsocial	Puzzle	Candy Crush, Bejeweled
	Platform	Super Mario Bros., Donkey Kong
	Adventure	Legend of Zelda, Tomb Raider

Adapted from Granic et al. [45••]

### EGE in Individual Psychotherapy

One of the earliest studies using EGE in behavioral therapy taught social skills to three autistic boys aged 17, 18, and 20 [46]. In a remarkably creative research design, the authors attempted to increase the frequency of appropriately initiated social interactions with neurotypical peers during classroom breaks in an outdoor courtyard of a public high school. The therapists trained the subjects to play the games and to approach and greet, offer to play, engage in reciprocal play, and signal the end of the interaction using three different items: a pack of gum, a Sony Walkman, and a handheld gaming device with Pac-Man or Galaxian. The Sony Walkman was the most effective adjunct in terms of time spent interacting by the students with mental retardation, and the video game was the most effective item for the higher-functioning student.

In a more recent case example, therapists used EGE in individual behavior therapy with three boys and one girl between the ages of 9 and 12 diagnosed with autism, who had fine motor skills adequate to manipulate the game controller [47]. The therapists’ role was to perform a set of interventions including video and in-vivo modeling. The treatment objective was to teach a common leisure skill using the Play Station 2 console to play Guitar Hero, a simple-social rhythm game. Subjects’ time on task increased significantly after the training and were able to play the game with at least 70 % accuracy, indicating that “training was effective in teaching young children with autism a generalized repertoire of an age-appropriate leisure skill, playing a video game” (p. 366).

One of the first reports of using EGE as an adjunct in individual talk therapy was a case study of a 7-year-old boy who presented with anxiety secondary to his parents’ divorce [2]. In addition to traditional psychotherapeutic methods including giving advice, providing emotional support, and encouraging behavior change, Allen [2] utilized “the experiences in the game and applied them to the patient’s real-life problems” (p. 332). Treatment outcomes included “self-confidence, a sense of mastery, more willingness to accept responsibility…and less stigma about having been in therapy” (p. 333). His choice of platform was a personal computer, and the game he chose was Ultima, a simple-nonsocial role-playing game (RPG).

Gardner [48] presented case studies on three children: a 5-year-old boy with disruptive behavior in school, a 10-year-old girl with anxiety, and a 7-year-old boy with an autism spectrum disorder. Gardner described one of his roles with the disruptive child as making, “(1) if-then statements for the moment (e.g., if you throw the controller, it’s cool off time) and (2) generalizing statements” (p. 668). The treatment objective was improved frustration tolerance and self-control. His roles with the remaining two children included having them watch him play, physically modeling play (“Therapist placed his fingers over J’s and together they played the game” p. 669), encouraging play by providing hints and prompts, and like Allen [2], discussing experiences in the game and generalizing them to others and self. The treatment objectives were improved coping skills for the anxious child and moving the child with developmental deficits from rigid behavior patterns to more flexible ones. The platform for all three children was a Nintendo console. The choice of game for the boys was Super Mario Brothers, a simple-nonsocial platform game; for the female client, Gardiner selected Jeopardy (simple-nonsocial puzzle game) to address performance anxiety in school and Legend of Zelda (simple-nonsocial adventure game) for separation anxiety.

Favelle [49] described using EGE “in a residential treatment facility for adolescents who were aggressive and who had poor social problem solving skills” (p. 152). Like Allen [2] and Gardner [48], he utilized experiences in the game and applied them to real life. Treatment objectives included engagement in therapy, improved problem solving, and insight. The platform used was a PC and two complex-nonsocial role playing games: The Wizard and the Princess and Alter Ego.

According to Friedberg [50•], cognitive-behavioral approaches using EGE are the latest stage in the evolution of CBT because of their appeal to clients, standardization, and availability. Computers and other digital technology facilitate the collection of research and assessment data. Research on game-based CBT (GB-CBT) may provide a good model for the use of and research on EGE. GB-CBT is an empirically supported approach to treatment of childhood trauma survivors that uses developmentally appropriate games (DAG), but not EG [51, 52]. GB-CBT uses games in group and individual therapy to facilitate the processing of traumatic memories, acquisition of social and emotional management skills, and group cohesion. Based on this example, future work on EGE should investigate empirically validated strategies and techniques, provide manuals with skill areas and menus of games, clearly defined goals and incentives, and experiential learning that provides opportunities for receiving corrective feedback. Clinicians can “establish a therapeutic environment, which promotes skill development, minimizes behavioral difficulties, and provides a positive, pleasant and motivating atmosphere” [53] (p. 1).

### EGE in Group Therapy

One of the first published accounts of using EGE for group therapy [49] used a group mystery game (Where in the World is Carmen Sandiego) to assess and enhance social skills through modeling. A more recent study using EGE in group therapy focused on social skills [54••]. Participants were nine boys, aged 7 to 11 years. Eight of the participants had ASD diagnoses, and one was diagnosed with ADHD and social skills deficits. A 10-week teaching interaction procedure similar to behavioral skills training was used, with the therapist and participants defining the targeted skill, followed by clinician modeling and participant dyads role-playing the targeted skills. The intervention targeted three skills: giving compliments, taking turns, and making a positive postgame comment. Therapists provided feedback and prompted participants to emit the targeted skills. The platform was a Nintendo Wii with Wii Sports, a simple-social sports game. The targeted skills significantly improved across baseline and training phases and generalized to different video games and actual sports. The authors noted that the “wide availability of such [electronic gaming] technology may allow for greater transportability of interventions across settings” (p. 303).

## Conclusions

In our survey of the research, electronic methods, including games and other types of computer-assisted therapies, have been shown to be equivalent but not superior in efficacy for a wide variety of medical health and mental health issues, in group, individual, and self-guided treatment. There is evidence that some electronic methods are more acceptable, enjoyable, or engaging than TAU and that greater therapist engagement may be associated with better outcomes. Methodological limitations include the predominance of quasi-experimental, pre-post designs, and case studies. Particularly for EGE, more exploration is needed into the efficacy of the use of commercially produced games, although it could be argued that games would simply be incorporated into an already evidence-based framework, such as CBT. The mechanisms of action for the use of video games in psychotherapy have been explored by various theorists and include attention, distraction, problem-solving, feedback, emotional expression, socialization, and exposure, but future studies should verify these actions’ relationship to outcomes. Granic et al. [45••] provide a strong framework that could facilitate game selection once parameters have been determined.

The area of therapeutic EG is promising, despite the need for more rigorous outcome studies. If, as has been suggested here, electronic methods produce similar results and are more acceptable than established treatments to clients, or to a subset of clients, their utility will be established based on ability to effectively serve a broader range of the population. EGP have the advantage of being tailored to specific client groups, diagnoses, and settings and are more standardized, but are relatively expensive to produce and must be specifically updated. When used at home, they are more specifically designed to retain their therapeutic effects. In order to benefit the client, the video games must be designed or chosen not only to serve a therapeutic purpose but also to be engaging and appropriate to the clients’ physical, cognitive, and emotional abilities as well as their age, gender, culture, and SES.

EGE, using off-the-shelf games, are easier and less expensive to acquire and update, provide a much larger set of options, and are generally more preferred by children and adolescents. EGE’s disadvantages include possible parental objections, the potential for unhelpful distraction, and the potential for game producers to eliminate features that facilitate the therapeutic aspects of play. For example, many of the Xbox and Xbox 360 games allowed up to four people to play on one console, which facilitated use in group therapy. The newer versions of many of the games require one console per player, and only a few allow two players per console. EGE require more engagement by the therapist, in general, which may have other benefits, and when played at home may produce positive effects, but are less targeted in this regard.

In a commentary on the adoption of the use of computers in psychotherapy by therapists, Barrett and Gershkovich [55•] raise six clinical, legal, and economic concerns: (1) the challenge of keeping up with advances in technology, (2) the potential detriment of computer-assisted technology to the therapeutic relationship, (3) time requirements for the therapist and compensation for that time in a significantly different format, (4) the potential for clients to access interventions not appropriate for them when online interventions are made openly available to the community, (5) implications of the decrease of face-to-face interaction between the client and therapist, including informed consent and assessment of dangerousness towards self or others, and (6) potential changes in content, maintenance, and confidentiality of records.

### Clinical Recommendations

#### Incorporate Technology in a Professional and Engaging way

The authors have a variety of computer-based and console-based games, some in the waiting room and some in practitioners’ offices. We also have handheld gaming devices like the Nintendo 3DS and tablets like the iPad and Nook in our individual offices. With respect to the waiting room, the consoles and games are kept in a separate room to minimize damage and prevent children from playing age-inappropriate games. In using EGE for groups, we use games that allow as many clients to participate as possible; for a single console, the best option at this time is the Wii U with up to five players at a time; for multiple consoles, three Xbox 360 consoles can accommodate up to 12 players at a time.

#### Select Games Based on Appeal to Clients and Their Potential for Meeting Therapeutic Goals

The games in our offices are generally purchased based on recommendations from clients. Clients may bring a game to session or, with parent permission, play one of our games. Manufacturer’s recommendations, parent feedback, and clinical expertise allow us to select games and tailor game play based on client age, cognitive functioning diagnosis, presenting problem, and level of skill. Games with graphic violent or sexual content should be avoided, and all content must be selected with an appreciation for not only the age but also the maturity of the client.

#### The Implementation by the Therapist Is Important to Producing Therapeutic Effects

Co-play with family members in family sessions and peers in groups allows for teaching social skills. Complex or simple social games would be used in these settings. Complex games matched to the developmental level provide adequate challenge and help build frustration tolerance. Simple social or nonsocial games can be used to break tension, provide distraction, and maintain adherence to therapy. Game play provides opportunities for problem solving, particularly with therapist-guided feedback. One of the authors devised a specific world in Minecraft which requires group members to engage in cooperative play in order to achieve a series of objectives.

#### Educate Parents About Risks and Protective Factors in Video Game Use

Research on the inappropriate use of EGE indicates that the amount of time gaming and the length of gaming sessions are related to the likelihood of social-emotional and educational problems, whereas playing with children and encouraging social play were key protective factors [56, 57]. Parents should be encouraged to play EG with their children to monitor their play for duration and content and to encourage them to play with friends, preferably in person rather than online. This is particularly important for clients for whom socialization and social skills are therapeutic goals. Parents should also be reminded that although games with sexual, violent, or otherwise objectionable content are commercially available, and despite research calling into question the extent of potential harm, in particular aggression and violence, they should be used with care—especially by children and adolescents [58].

In summary, EG and other technologies can provide effective, efficient, and appealing interventions for a variety of clients. The psychotherapeutic use of EGE as an adjunct to individual or group therapies has a long tradition that is consistent with play and group therapy in general, although considerably less empirically researched than the latter. EGP is a growing area with a large number of games with varying levels of specificity. With smart devices that are carried with us, using the Internet, e-mail, and social media, we are able to gather and disseminate information in ways that are less time-consuming, more interesting, and more readily available. With advanced graphic and audio-visual performance, we are able to create virtual multisensory worlds in order to expose clients to important stimuli in a safe manner without limiting ourselves to the physics or logistics of the real world. The use of EG may allow us to engage our clients in a way that is not only more appealing to many clients, especially children and adolescents, but also with demonstrated efficacy.
